# Janus kinase signaling as risk factor and therapeutic target for severe SARS‐CoV‐2 infection

**DOI:** 10.1002/eji.202149173

**Published:** 2021-03-22

**Authors:** Farzan Solimani, Katharina Meier, Kamran Ghoreschi

**Affiliations:** ^1^ Department of Dermatology, Venereology and Allergology Charité–Universitätsmedizin Berlin Berlin Germany

**Keywords:** Janus kinase, JAK inhibitors, SARS‐CoV‐2, severe COVID‐19, cytokine storm

## Abstract

Cytokine signaling, especially interferon (IFN) signaling is closely linked to several aspects of severe acute respiratory syndrome coronavirus 2 (SARS‐CoV‐2) infection. During initial SARS‐CoV‐2 infection, symptomatic patients present with impaired type I/III IFN‐mediated antiviral responses. Interestingly, IFNs regulate the cellular entry receptor for SARS‐CoV‐2 on epithelial and endothelial cells. As reported recently, critically ill COVID‐19 patients show genetic polymorphisms in one IFN receptor gene (*IFNRA2*) and in a gene locus near the Janus kinase (JAK) *TYK2*, which is key for IFN, interleukin (IL)‐12 and IL‐23 signaling, and T helper (Th) 1/Th17 cell‐mediated antiviral immune responses. In the advanced stage of the disease, critically ill COVID‐19 patients develop a cytokine storm where many inflammatory mediators using the JAK/STAT signaling pathway such as IL‐6, IFN‐γ, the granulocyte colony‐stimulating factor (G‐CSF) or IL‐2, and chemokines result in an influx of macrophages and neutrophils damaging the lung tissue. The knowledge on the cytokine and JAK/STAT signaling pathways in severe COVID‐19 disease explains the promising first results with JAK inhibitors like baricitinib, which not only dampen the inflammation but in the case of baricitinib also affect virus replication and endocytosis in target cells. Here, we summarize the current immunological associations of SARS‐CoV‐2 infection with cytokine signaling, the JAK/STAT pathway, and the current clinical stage of JAK inhibitors for improving severe COVID‐19 disease.

## Graphical abstract

The interferon receptor and the JAK/STAT signaling pathway are genetically and functionally linked to severe diseased COVID‐19 patients. JAK inhibitors may affect viral endocytosis and provide anti‐inflammatory effects in these patients. This modern class of drugs may help to prevent the cytokine and chemokine storm in SARS‐CoV‐2‐infected patients with severe disease and reduce lung influx with neutrophils and macrophages.

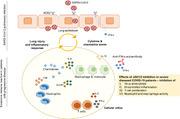

## Introduction

**Figure 1:**
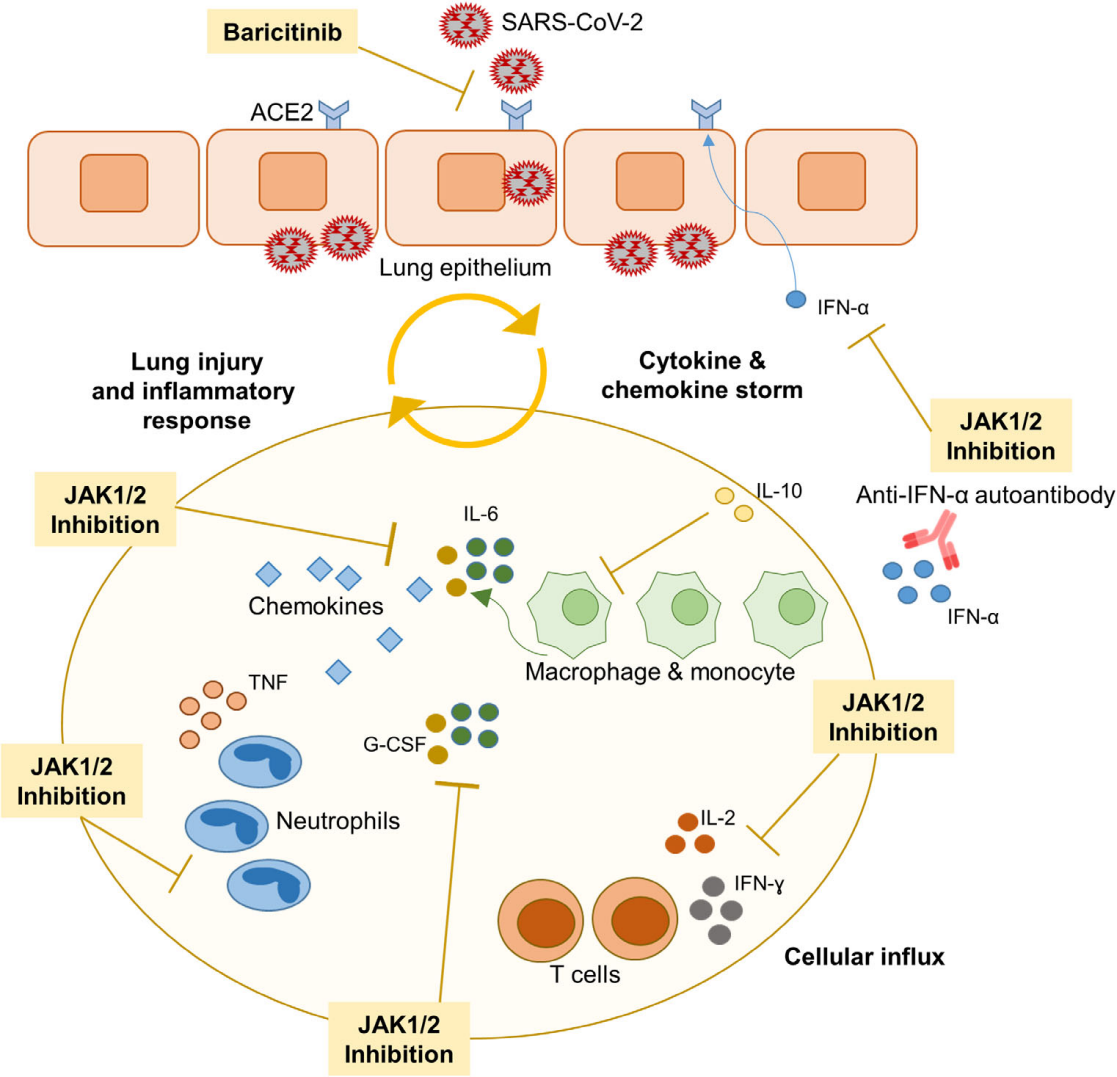
Importance of cytokine signaling through Janus kinases (JAK) during SARS‐CoV‐2 lung infection. Viral endocytosis in lung epithelial cells begins through the ACE2 receptor, which is regulated by IFN signaling. The initial antiviral response mediated by type I/III IFNs and downstream JAK/STAT signaling is impaired in severely ill patients. In these patients with progressive disease, SARS‐CoV‐2 infection triggers an excessive immune reaction with release of multiple pro‐inflammatory cytokines such as IL‐2, IL‐6, granulocyte colony stimulating factor (G‐CSF) that signal through the JAK/STAT pathway, and others like TNF. Infiltrating macrophages and neutrophils contribute to lung injury. A genetic susceptibility with polymorphisms in or near the genes for IFNAR2 and TKY2, respectively, and, the development of autoantibodies against IFNs favor the inflammatory profile in severely ill patients. The JAK1/JAK2 inhibitor baricitinib exerts antiviral effects by hampering virus endocytosis and profound anti‐inflammatory effects in severely ill patients by blocking cytokine signaling.

Twelve months after the outbreak of first cases of the COVID‐19 pandemic in the Hubei province of China [1], the severe acute respiratory syndrome coronavirus 2 (SARS‐Cov‐2) is still stressing societies and healthcare systems around the world. In the end of 2020, the first vaccines against the novel coronavirus have been approved by the U.S. and European authorities [2, 3]. Albeit many regions of the world are currently experiencing an increase in infections and deaths, the impact of the vaccines on this pandemic will be tangible earliest in several months. We are still in a threatening situation where we need to improve our understanding on COVID‐19 disease and therapeutic management to refine the current standard of care. So far, several different therapeutic approaches including antiviral drugs, anti‐inflammatory agents, immunotherapies, or a combination of such therapies have been tested [4]. One important support for clinicians emerges from immunological studies aiming to characterize the inflammatory response in infected patients. While the majority of patients display mild to moderate respiratory problems, a subgroup—especially elderly individuals and/or those with certain comorbidities—develop severe respiratory distress and systemic inflammation, where an impaired virus specific T‐cell answer and the production of inflammatory cytokines eventually results in a cytokine storm syndrome [5, 6, 7]. This phenomenon is not based on viral load but rather on the individual immune answer to the virus. At this stage, patients show an excessive expression of inflammatory cytokines (tumor necrosis factor [TNF], interleukin [IL]‐1B, IL‐6, IL‐7, IL‐8, IL‐10, IL‐12, IL‐23, granulocyte colony stimulating factor (G‐CSF) and interferon [IFN]‐γ), and associated chemokines (chemokine (CC motif) ligand (CCL)2, CCL8, CCL20, chemokine (CXC motif) ligand (CXCL)1, CXCL3, CXCL5, CXCL6, CXCL2, and CXCL16) (Fig. 1) [5, 8, 9]. In this review, we aimed to summarize some important facts on the JAK/STAT signaling pathway during SARS‐CoV‐2 infection and the potential benefit of JAK inhibitors on the clinical outcome in severe diseased patients.

## Targeting single cytokines as immunotherapy for COVID‐19

Due to the knowledge on the cytokine pattern in patients infected with SARS‐CoV‐2, the current drug interventions for hospitalized patients requiring supplemental oxygen or ventilation comprise a combination of antiviral agents like remdesivir together with anti‐inflammatory drugs like dexamethasone. The prominent production of IL‐6 as induced by SARS‐Cov‐2 led to the use of monoclonal antibodies directed against the IL‐6 receptor in hospitalized patients very early on. In the meanwhile, first phase 3 trials with tocilizumab and sarilumab (both blockers of the IL‐6 receptor) in the setting of severe COVID‐19 pulmonary disease have been published with contradictory results. However, some patients may benefit from IL‐6 blockade [10, 11, 12]. Yet, immunotherapies targeting a broader range of cytokines or their signaling events may offer better strategies. Remarkably, patients with the chronic IL‐17/IL‐23‐dominated inflammatory skin disorder psoriasis receiving immune treatments (i.e., anti‐IL‐17/anti‐IL‐23 monoclonal antibodies or phosphodiesterase 4 inhibitor) seemingly show a lower risk for adverse outcomes during SARS‐CoV‐2 infection [13, 14].

## Importance of type I/III IFN responses during SARS‐CoV‐2 infection

Severely affected COVID‐19 patients seem to present with an insufficient induction of type I and type III IFNs during initial illness [15, 16]. This is in contrast to the situation seen in patients with influenza infections, where a sufficient IFN‐dominated antiviral response typically appears upon contact of the host's pattern recognition receptors to viral RNA [5, 8]. Of note, a subgroup of patients with life‐threatening COVID‐19 pneumonia shows neutralizing autoantibodies against IFNs at the onset of critical disease [17] (Fig. 1). On the other hand, type I IFNs limit excessive virus‐dependent inflammatory lung injury by inducing IL‐10, as observed in experimental influenza infection [18]. Finally, type I IFNs critically regulate the expression of the cellular entry receptor for SARS‐Cov‐2 (ACE2) in airway epithelial cells, enterocytes of the small intestine, and endothelial cells [19, 20]. Presumably, this regulation is more critical during the initial infection phase of the disease. Thus, type I/type III IFN receptor subunits (IFNAR1/IFNAR2 or IFNλR1/IL10R2) and their associated signaling proteins of the Janus kinase (JAK) family (JAK1 and TYK2) are of special relevance in COVID‐19 patients. Genetic findings further underline the relevance of cytokine receptor signaling pathways in viral infections and COVID‐19 disease. Single cases of human IFNAR1/IFNAR2 deficiencies report from fundamental complications after vaccination with live attenuated viruses [21, 22]. Likewise, TYK2‐deficient patients show impaired cellular responses to IL‐12, IL‐23, and type I IFNs and present higher susceptibility for viral infections [23]. Importantly, a genome‐wide association study conducted in 2244 critically ill COVID‐19 patients unraveled significant associations on chr21q22.1 (rs2236757) in the gene locus of IFNAR2 and on chr19p13.3 (rs74956615) near the gene encoding TYK2 [24]. Here, Pairo‐Castineira et al. further analyzed these findings by Mendelian randomization, finding an association between *IFNAR2* low expression, *TYK2* high expression, and severe disease course [24].

## JAK inhibitors for treatment of severe COVID‐19

Based on this pivotal role for type I and III IFNs, therapeutic approaches with IFN‐α or IFN‐β have been tried, though they were of limited benefit in COVID‐19 patients, indicating that the early antiviral and anti‐inflammatory effects of type I IFN cytokine administration are not sufficiently protective. As suggested very early during the pandemic, a broader inhibition of the cytokine storm may be of greater relevance [25, 26]. Inhibitors that inhibit JAKs exert potent anti‐cytokine effects and dampen the signals of multiple factors that are increased in SARS‐Cov‐2‐infected individuals (e.g., IL‐2, IL‐6, IL‐7, IFN‐α, IFN‐γ, and G‐CSF). Such inhibitors should target at least JAK1, preferentially in combination with JAK2 or even TYK2. Initial positive results with the JAK1/JAK2 inhibitor ruxolitinib were reported from an Italian study. A small cohort of COVID‐19 patients with severe pulmonary disease (*n* = 34) was treated with ruxolitinib. As published, amelioration of pulmonary function was observed in about 85% of the patients [27]. Another JAK inhibitory compound that has been extensively tested in the setting of SARS‐Cov‐2 is baricitinib, a JAK1/JAK2 inhibitor with lower inhibitory potency toward TYK2, which is in clinical use for the treatment of patients with rheumatoid arthritis or atopic dermatitis and is under investigation for a large number of inflammatory and autoimmune diseases [28, 29, 30]. Published data from a double‐blind, randomized, placebo‐controlled trial in 1033 hospitalized COVID‐19 patients demonstrated that baricitinib in combination with remdesivir resulted in reduced hospitalization period and accelerated recovery time in critically ill patients receiving high‐flow oxygen or noninvasive ventilation compared to remdesivir alone [31]. Thus, inhibition of multiple cytokines’ signaling seems to be more promising than manipulating the action of single cytokines. In November 2020, baricitinib received an emergency use authorization by the FDA for the treatment of severely ill COVID19 patients. While the immunological effects achieved with baricitinib may also be valid for some other JAK inhibitors, some off‐target effects seem to be unique for baricitinib. As suggested by bioinformatic approaches and confirmed by in vitro models and kinase assays, baricitinib could reduce cellular infection by blockade of numb‐associated kinase members that are implicated in receptor‐mediated viral endocytosis [32, 33]. Baricitinib shows nanomolar affinity for the numb‐associated kinase family members’ AP2‐associated protein kinase 1 (AAK1) and cyclin G‐associated kinase (GAK) and reduces the viral load in liver spheroids infected with SARS‐CoV‐2 [33]. Like other clinically advance staged JAK inhibitors, baricitinib potently suppresses the intracellular signaling of cytokines such as IL‐6, IFN‐α, or IFN‐γ in vitro [34] and in a rhesus macaque model of SARS‐CoV‐2 infection, where JAK1/JAK2 blockade also showed a significant decrease in macrophages and neutrophils infiltrating the lungs [35].

Baricitinib along with other JAK inhibitors may be a better strategy than dexamethasone. The use of baricitinib in COVID‐19 patients was not associated with an increase in thromboembolic events and infection‐related adverse events were fewer than in the placebo group [31]. This was not expected, since virus reactivation (i.e., herpes zoster) is normally observed in patients under JAK inhibitor treatment. Of note, no increase in thromboembolic events was observed, although such events are frequent in SARS‐CoV‐2‐infected patients and appear in patients receiving JAK inhibitors for autoimmune diseases [36]. While clinical trials with baricitinib for COVID‐19 patients are ongoing, the efficacy and safety of other JAK inhibitors alone or in combination with other agents are under clinical investigation in the setting of SARS‐CoV‐2‐infected patients (Table 1). Most of these inhibitors tested are either selective for JAK1/JAK2 (baricitinib, ruxolitinib) or JAK1/JAK3 (tofacitinib), given orally and approved for other diseases than COVID‐19. Interestingly, one pan‐JAK inhibitor (TD‐0903), originally developed as topical JAK inhibitor for preventing graft rejection in patients with lung transplantation, is currently being tested as an inhalation formulation. According to *clinicaltrials.gov*, none TYK2 inhibitor is currently tested, although the genetic association of severe ill COVID‐19 populations with a region near the TYK2 gene has been recently reported [24]. However, the functional consequence of this polymorphism is not clear yet. The successful management of severe ill COVID‐19 patients is still of highest priority. This pandemic with >80 million infections has already resulted in the loss of more than 2.5 million lives worldwide and wide‐scale vaccination programs just started in designated countries and will take long time. The understanding of the role of cytokine signaling and virus behavior in SARS‐CoV‐2 infection helps to establish effective treatments. Immune‐regulating JAK inhibitors are among the most promising strategies, although this new class of drugs was developed for myeloproliferative and autoimmune diseases and not for combating viral diseases [37].

**Table 1:** Current trials with Janus kinase inhibitors in the management of severe acute respiratory syndrome coronavirus 2 (SARS‐CoV‐2) infected patients

JAK inhibitor	Target	COVID‐19 patient population	Administration	Trial phase	NCT number
Ruxolitinib	JAK1/2	ARDS	Oral	2/3	NCT04477993
Ruxolitinib	JAK1/2	ARDS with ventilation	Oral	3	NCT04377620
Baricitinib	JAK1/2	Moderate to Severe	Oral	2	NCT04321993
Tofacitinib^a,1^	JAK1/3	Interstitial pneumonitis	Oral	2	NCT04390061
TD‐0903	Pan‐JAK	Symptomatic acute lung injury	Inhalation	2	NCT04402866
Ruxolitinib	JAK1/2	Pneumonia	Oral	2	NCT04334044
Ruxolitinib^b,2^	JAK1/2	Severe stages 2b/3	Oral	3	NCT04424056
Ruxolitinib	JAK1/2	Pneumonia	Oral	NA	NCT04331665
Tofacitinib	JAK1/3	Pneumonia	Oral	2	NCT04332042
Baricitinib^c^	JAK1/2	Mild to moderate	Oral	2/3	NCT04320277
Ruxolitinib	JAK1/2	Safety & Efficacy	Oral	2/3	NCT04348071
Baricitinib	JAK1/2	Safety & Efficacy	Oral	2/3	NCT04340232
Baricitinib	JAK1/2	Pneumonia	Oral	2	NCT04399798
Ruxolitinib^3^	JAK1/2	Pneumonia	Oral	1/2	NCT04581954
Baricitinib^d,4^	JAK1/2	Hospitalized Patients	Oral	3	NCT04640168
Ruxolitinib	JAK1/2	ARDS	Oral	2	NCT04359290
Ruxolitinib^b,5^	JAK1/2	Pneumonia	Oral	2	NCT04366232

In combination with hydroxychloroquine^a^, anakinra or tocilizumab^b^, ritonavir^c^, and remdesivir^d^.

Compared to treatment group with hydroxychloroquine alone^1^, standard of care, anakinra and tolicizumab alone^2^, to standard of care and fosfatinib^3^, to dexamethasone with baricitinib^4^, to anakinra alone^5^.

## Conflict of interest

The authors declare no commercial or financial conflict of interest.

**Abbreviation**
SARS‐Cov‐2: severe acute respiratory syndrome coronavirus 2
